# Pediatric cataracts of different etiologies contain insoluble, calcified particles

**DOI:** 10.3389/fopht.2023.1213359

**Published:** 2023-07-07

**Authors:** Peter J. Minogue, Sarah H. Rodriguez, Viviana M. Berthoud, Eric C. Beyer

**Affiliations:** ^1^Department of Pediatrics, University of Chicago, Chicago, IL, United States; ^2^Department of Ophthalmology and Visual Science, University of Chicago, Chicago, IL, United States

**Keywords:** cataract, calcium precipitation, biomineralization, lens, pediatric

## Abstract

Our recent studies in mice suggest that a crucial event for the development of cataracts is the formation of calcium-containing deposits. To examine the generality of pathologic mineralization as a novel mechanism of cataract formation, we analyzed lens material from different human cataract surgeries. Human lens material was obtained from routine cataract surgeries performed on three patients with dense, white cataracts: a 10-month-old with congenital cataracts, a 9-year-old with a uveitic cataract, and a 17-year-old with a traumatic cataract. The aspirated material from the cataract surgeries contained insoluble material that could be isolated by centrifugation. Many particles within the insoluble fraction stained with Alizarin red, a dye that stains insoluble calcified material. The appearance of these human insoluble, Alizarin red-stained particles was similar to some of those detected in homogenates from cataractous mouse lenses. These results support the hypothesis that pathologic mineralization may have a mechanistic role in the formation of cataracts of different etiologies.

## 1 Introduction

Although less common than in adults, pediatric cataracts are the leading cause of childhood blindness worldwide (1, 2). While they may result from a variety of different primary etiologies, cataracts in children share several pathologic features. Cataractous lenses exhibit damage to lens cell proteins and lipids. Biochemically, this damage can include oxidation, crosslinking, denaturation, aggregation, proteolysis/cleavage, misfolding, and other kinds of modifications (3, 4). Disturbances of ionic homeostasis, including large elevations of calcium concentrations, are also observed in many cataractous lenses (5, 6). These biochemical changes are not necessarily identical in all patients and their relative importance for cataract severity is not well established.

Recent studies performed in our laboratory and others using microscopy and micro-computed tomography to study the lenses of mice that develop cataracts due to different genetic abnormalities have shown that the cataracts contain calcium precipitates that localize to the same region as the cataract (7–10). In connexin mutant lenses and the lenses of a mouse with a mutation of γC-crystallin, we used infrared microspectroscopy to identify the mineral in these precipitates as calcium phosphate in the form of apatite (10, 11). Radiographic studies have detected calcium-containing mineral also in canine cataracts (12). Several previous papers have suggested that different types of cataracts in people contain insoluble calcium salts (13–17). These findings suggest that precipitation of calcium ions (biomineralization) might be a general phenomenon in cataracts (18).

We undertook the present study to test the generality of calcified particle formation in human cataracts of different etiologies. We performed microscopic examination of the insoluble material from cataract extractions after staining it with Alizarin red, a dye commonly used to identify and localize calcium deposits in tissues, including bone and teeth.

## 2 Methods

### 2.1 Subjects

Patients seen at Comer Children’s Hospital for cataract extraction were enrolled prospectively. Parents provided informed consent. Assent was obtained from children aged seven years or older unless the child's decision-making capacity was impaired. Protocols were approved by the University of Chicago Institutional Review Board (#17-1679). All studies were conducted in accordance with the Declaration of Helsinki guidelines.

### 2.2 Alizarin red staining of lens material and microscopy

**Figure 1:**
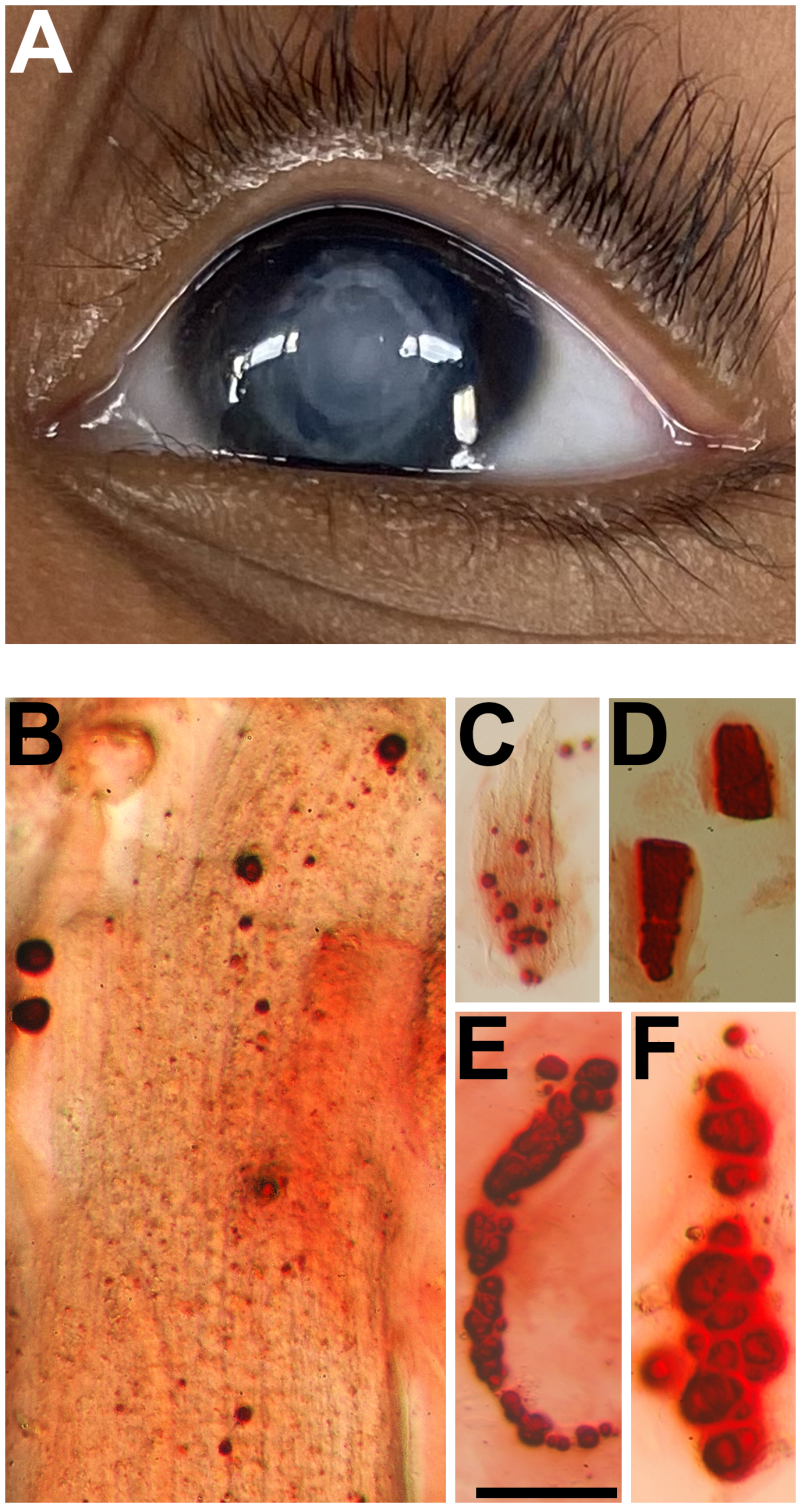
**(A)** Pre-operative external photograph of the left eye of a 10-month-old girl with bilateral congenital cataracts. **(B–F)** Photomicrographs showing the Alizarin red-stained particles in the insoluble material extracted from this lens. Bar, 50 µm for **(B, D, F)** and 100 µm for **(C, E)**.

**Figure 2:**
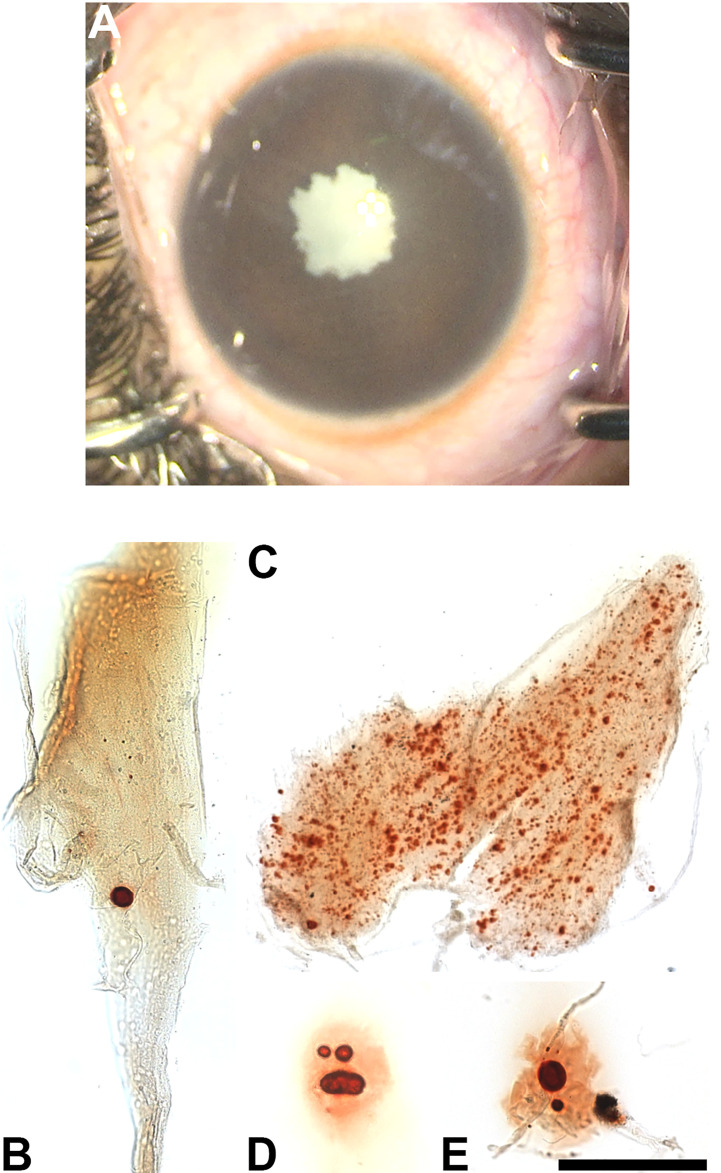
**(A)** Pre-operative photograph of the right eye of a 9-year-old girl with a uveitic cataract. **(B–E)** Photomicrographs showing the Alizarin red-stained particles in the insoluble material extracted from this lens. Bar, 161 µm for **(B, C, E)** and 81 µm for **(D)**.

Lens material aspirated from cataract surgery, which would otherwise be discarded, was analyzed. All material collected during cataract surgery (including irrigation fluid) was combined (total volume 50 - 150 ml) and centrifuged at 16000 g for 10 min at 4°C. The resulting pellet was resuspended and fixed for 15 min at room temperature in 4% paraformaldehyde in PBS, pH 7.4 (total volume 0.5 ml) to enhance preservation of cells and tissue pieces in the insoluble fraction. After fixation, the lens material was centrifuged and the pellet was resuspended in PBS, pH 7.4. The resuspended material was mixed with an equal volume of 2% Alizarin red (filtered) in water (pH 4.1-4.3) on a glass slide (11). Images from the specimens were acquired using 10X or 20X objectives with a Nikon DIAPHOT inverted microscope (Nikon Instruments Inc., Melville, NY) and a Nikon D70 digital camera as described previously (11) or using 10X, 20X or 40X Plan-Apochromat objectives in a Zeiss Axioplan 2 microscope (Carl Zeiss, München, Germany) equipped with a Zeiss AxioCam digital camera using Zeiss AxioVision software. The longest dimensions of the Alizarin red-stained particles were determined using ImageJ (19). For images including many particles in different focal planes, multiple photomicrographs of the same field were combined using the ImageJ Extended Depth of Field (EDF) plugin; EDF-combined images are shown in **Figures 1B**, **2C**.

## 3 Results

We studied three pediatric patients with cataracts of different etiologies (**Table 1**).

**Table 1:** Patient characteristics.

	Patient 1	Patient 2	Patient 3
Age	10 months	9 years	17 years
Sex	Female	Female	Female
Cataract etiology	Congenital/genetic	Uveitis	Trauma
Time to apparent white cataract	~3 months	1 month	~1 month
Affected eye	Both	Right	Right

### 3.1 Patient 1 congenital/genetic cataracts

A 10-month-old girl presented as a referral from her pediatrician due to poor red reflex in both eyes that had been first noticed by the mother at about 7 months. She was found to have bilateral white cataracts (**Figure 1A**) and poor fixation. There was no view to the retina due to the dense white cataracts, but posterior segment ultrasonography was unremarkable.

Her newborn screen had been negative, and she had passed a hearing screen shortly after birth. The genetics consultant did not recognize any dysmorphic features on exam and did not suggest a syndromic diagnosis. Microarray testing showed single copy interstitial loss of chromosome 8q24.3. The patient’s younger sibling carries the same mutation and has milder cataracts that are not yet visually significant.

The patient underwent lensectomy with intraocular lens placement, limited anterior vitrectomy, and posterior capsulectomy in both eyes. After removing the cataracts, examination of her optic nerve and retina were unremarkable. Her nystagmus has continued with some intermittent visual interest likely due to a combination of amblyopia and cortical visual impairment.

### 3.2 Patient 2 uveitic cataract

A 9-year-old girl presented with poor vision in the right eye of unknown duration. The family had noticed a white pupil on the right side 1 month prior to presentation to the emergency room. She had no history of trauma. A normal eye exam was documented on optometry records from 5 years prior. There was no family or personal history of autoimmune conditions. Past medical history and review of systems were negative.

On examination, her vision was 20/20 in the left eye and hand motions in the right eye. Pressure was normal in both eyes. The left eye examination was unremarkable with no signs of inflammation or cataract. In the right eye, she was found to have a dense white cataract (**Figure 2A**) with 360 degrees synechiae. The anterior segment was otherwise quiescent. Posterior segment ultrasonography revealed some scattered hyper-echoic opacities in the vitreous, hyper-echoic signal over the nerve, but attached retina.

Rheumatology consultation and serologic evaluation showed no evidence for an underlying autoimmune condition. The patient was diagnosed with idiopathic uveitis.

The subject underwent cataract surgery with synechiolysis and intraocular lens placement with intracameral steroids. Post-operatively, she was found to have optic nerve edema and cystoid macular edema. She was placed on topical and oral steroids, and her disc and macular edema resolved. Best corrected visual acuity is 20/50. She has been tapered off steroids, and the eye has remained quiet.

### 3.3 Patient 3 traumatic cataract

**Figure 3:**
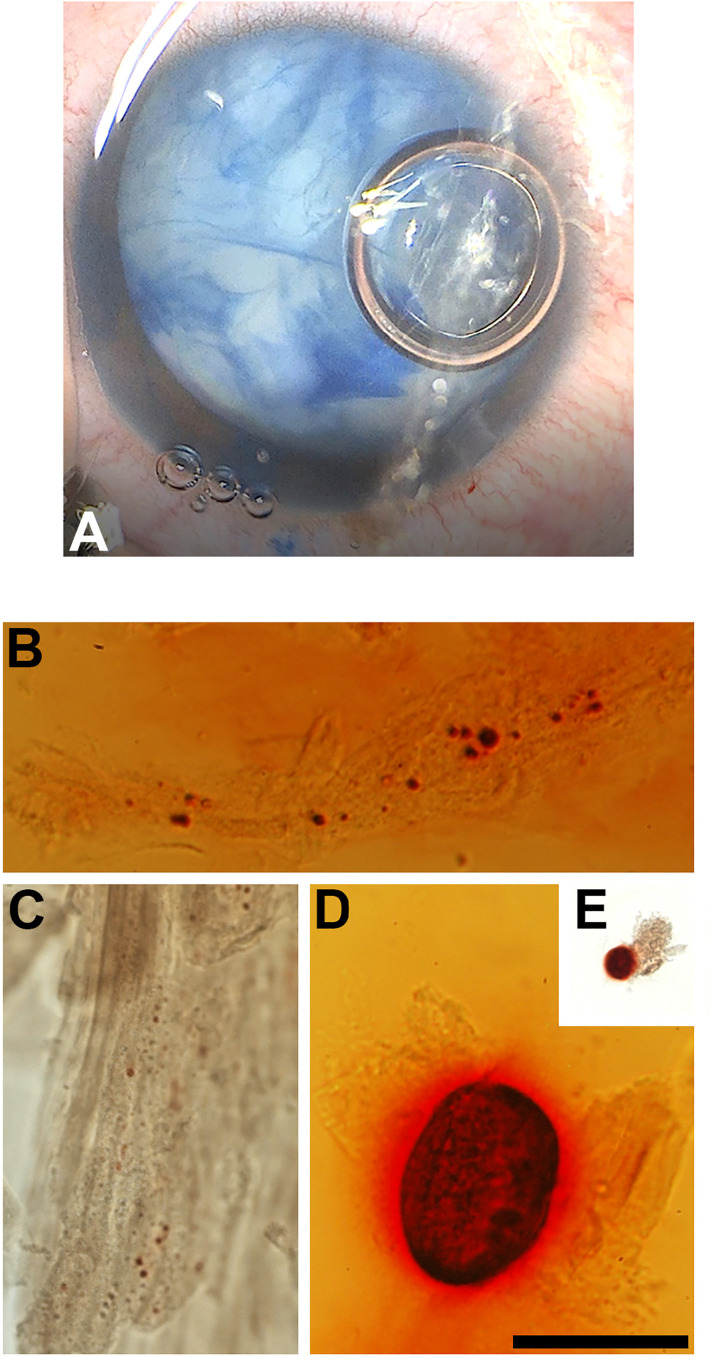
**(A)** Intra-operative photograph of the right eye of a 17-year-old girl with a traumatic cataract, after staining of the anterior capsule with trypan blue. **(B–E)** Photomicrographs showing the Alizarin red-stained material in the insoluble material extracted from this lens. Bar, 50 µm for **(B, D)** 41 µm for **(C)** and 161 µm for **(E)**.

A 17-year-old girl with the 13q deletion syndrome, developmental delay and self-inflicted trauma presented with a white, traumatic cataract in the right eye (**Figure 3A**). The patient had a significant past medical history (severe intellectual disability, hearing impairment, dependence on gastrostomy feeding, type 2 diabetes, asthma, and atrial and ventricular septal defects), but she had no previously diagnosed eye problems. The mother reported the new development of a white pupil within a few weeks to a month prior to presentation. Visual acuity could not be assessed due to lack of patient cooperation. There was no view of the retina before surgery, but ultrasound was reassuring. The left eye examination was unremarkable.

She underwent lensectomy with intraocular lens placement in the right eye. Her retina was subsequently visualized and appeared normal. A laser retinopexy was recommended for both eyes to decrease the chance of a traumatic retinal detachment.

### 3.4 Examination of insoluble material

The insoluble fractions from the lenses of all three patients contained many particles that stained with Alizarin red (**Figures 1B–F**, **2B–E**, **3B–E**). The Alizarin red-stained objects varied widely in size: 0.5-84 µm for patient 1, 1.5-32 µm for patient 2, and 0.4-57 µm for patient 3. Some lens fiber cells were associated with large numbers of very small particles (**Figures 1B**, **2B, 2C**, **3C**). Some of the larger particles had distinct, sharp linear edges and appeared refractile, suggestive of crystals (**Figures 1D**, **2D**, **3D**).

## 4 Discussion

We found Alizarin red-stained insoluble particles in all cases within this series of three pediatric patients with cataracts of diverse etiologies, suggesting that biomineralization (calcification of biological material) may be a general occurrence in pediatric cataracts. Many of the particles were discrete with limited background staining of most cellular material and debris in the samples, implying the specificity of our staining. We have not performed “control” experiments to study insoluble material from the clear lenses of normal children for comparison with the cataractous material. However, based on extrapolating our previous findings that Alizarin red-stained particles are found in the lenses of mice with cataracts but not in the lenses of their wild type littermates (10, 11), we suspect that such stained particles are uniquely associated with cataractous lenses.

Alizarin red has been widely used as a histochemical stain to identify calcified material in tissues (20, 21) or in body fluids (22). This dye is not absolutely specific for calcium, since it can also react with salts of other cations (23). However, calcium ions are more abundant in tissues than the other cations (e.g., copper, barium, zinc, lead, iron, nickel and cobalt). In mouse models that develop cataracts, an increase in the intracellular concentration of Ca^2+^ > 1 μM is a common finding (6), making it potentially possible to exceed the *K_sp_* for some calcium salts in these lenses or to favor the deposition of calcium on a scaffold of macromolecules containing negative charges. In our studies of mutant mice, we found mineral in their cataractous lenses and identified it as calcium phosphate in the form of apatite (10, 11). Therefore, it is likely that calcium ions comprise the cation in the Alizarin red-stained insoluble material that we found in pediatric cataracts.

Maintenance of lens cell homeostasis is facilitated by an internal circulation of ions, small molecules and water, which is driven by the regional distribution and activities of ion channels, transporters, and exchangers (24). Ions enter the lens at the anterior and posterior poles and move towards the center through the extracellular spaces. Ions are driven into fiber cells by their electrochemical gradients. Ions move outward through the cytoplasm of fiber cells and across cell boundaries through gap junction channels. Once ions reach the epithelial cells on the lens surface, they are transported out of the lens by epithelial ion pumps, transporters and exchangers. The movement of water is coupled to the circulation of ions.

Cellular Ca^2+^ is highly regulated in the lens. In the epithelial cells, the intracellular Ca^2+^ concentration is maintained at ~100 nM (25, 26). In fiber cells, it is somewhat higher, reflecting a gradient of calcium ions that increases approaching the center of the lens. Regardless of cell type, the intracellular concentration of Ca^2+^ is thousands of times less than the extracellular Ca^2+^ concentration of ~1.3 mM in the aqueous humor (5, 27). Thus, the electrochemical gradient will drive extracellular Ca^2+^ that enter the lens into fiber cells. Impairment of the lens circulation will lead to increases in the intracellular concentration of Ca^2+^, which can interact with anions in the cells (including free phosphate) and form insoluble particles.

The sequence of events leading to biomineralization may differ in subjects that develop cataracts due to different primary etiologies. The initial event may be precipitation of insoluble calcium-containing salts resulting from disturbances in ionic homeostasis or it might be damage to lens components that act as a matrix for the deposition of calcium ions. Each of these sequences may explain the formation of mineralized particles in the different cases studied in this report.

The lens circulation of ions and water is disrupted in many different kinds of cataracts (28). Lens calcification has been observed in mouse cataracts caused by mutations of channel proteins with varied functions (7–9, 11, 29) and cataracts caused by mutations of other lens proteins associated with secondary impairment of the lens circulation (10). We do not know the gene(s) responsible for the congenital cataracts in our series, but a primary disturbance of lens ionic homeostasis might be possible. In the traumatic cataract, direct impact on the globe causes lens damage and cellular disruption (30), exposing negative charges in proteins and other molecules that could readily bind calcium ions. Cataract formation in patients with uveitis is usually attributed to uncontrolled and sustained inflammation (or prolonged use of steroids which was not the case in our patient) (31). It is likely that pathologic calcification followed the inflammatory damage to lens cells.

Formation of calcified material in the lens requires interaction of Ca^2+^ with negatively charged ions or molecules. The extracellular pool would provide the main source for Ca^2+^ accumulation inside lens cells and subsequent mineralization in the many kinds of cataracts that develop when the lens circulation is disrupted (28). Mitochondria and the endoplasmic reticulum hold substantial stores of calcium ions that may contribute to mineralization in some forms of cataracts. Although these organelles are normally only present in epithelial and differentiating fiber cells, damage to cortical cells might result in release of their intraorganellar calcium. The anions (including phosphate and possibly proteins or lipids) are all major constituents of cells and their cytoplasm that may become available for interaction with Ca^2+^ in lenses subjected to insults or damage.

Regardless of the mechanistic sequence of events, biomineralization within the lens would have a major impact on vision (beyond protein damage and aggregation). The calcium-containing particles would scatter or block transmission of light onto the retina. It is not yet clear how the abundance of calcified material relates to the volume or severity of cataracts. All of the patients in our series had dense white cataracts of unknown duration. In the two older patients, the parents had only noticed the white pupil for about one month prior to presentation, while the congenital cataract may have been significant for as little as three months. These results suggest that mineralization may occur relatively early in some (or perhaps all) cataracts.

Taken together, our data suggest that development of a treatment that prevented biomineralization in the lens could be beneficial by interfering with the development or progression of cataracts.

## Data availability statement

The original contributions presented in the study are included in the article. Further inquiries can be directed to the corresponding author.

## Ethics statement

The studies involving human participants were reviewed and approved by University of Chicago Institutional Review Board. Written informed consent to participate in this study was provided by the participants’ legal guardian/next of kin. Written informed consent was obtained from the minor(s)’ legal guardian/next of kin for the publication of any potentially identifiable images or data included in this article.

## Author contributions

PM, SR, VB, and EB conceived the project. SR conducted the clinical evaluations of all patients and performed the surgeries. PM performed the microscopy experiments. PM and VB analyzed the data and prepared the figures. All authors contributed to the article and its writing, and approved the submitted version.
